# Comparison of Microwave-Assisted and Conventional Hydrodistillation in the Extraction of Essential Oils from Mango (*Mangifera indica* L.) Flowers

**DOI:** 10.3390/molecules15117715

**Published:** 2010-10-29

**Authors:** Hong-Wu Wang, Yan-Qing Liu, Shou-Lian Wei, Zi-Jun Yan, Kuan Lu

**Affiliations:** School of Chemistry & Chemical Engineering, Zhaoqing University, Zhaoqing 526061, China

**Keywords:** *Mangifera indica* L., essential oil, microwave-assisted hydrodistillation, GC-MS

## Abstract

Microwave-assisted hydrodistillation (MAHD) is an advanced hydrodistillation (HD) technique, in which a microwave oven is used in the extraction process. MAHD and HD methods have been compared and evaluated for their effectiveness in the isolation of essential oils from fresh mango (*Mangifera indica* L.) flowers. MAHD offers important advantages over HD in terms of energy savings and extraction time (75 min against 4 h). The composition of the extracted essential oils was investigated by GC-FID and GC-MS. Results indicate that the use of microwave irradiation did not adversely influence the composition of the essential oils. MAHD was also found to be a green technology.

## 1. Introduction

Mango (*Mangifera indica* L.) is one of the most important and popular tropical fruits, due mainly to its delicious flavor and nutritional value [1]. In various parts of the world, several hundreds of cultivars that have markedly varying flavor characteristics are grown [2]. Thus, numerous studies have been conducted on mango flavor composition. The volatile constituents of mango have also been extensively investigated. In past decades, more than 300 volatile components have been identified from several varieties of mango [3,4,5,6,7,8,9,10,11,12,13,14]. These compounds are free forms or glycosidically bound compounds [15,16,17,18,19]. In these varieties, considerable variation in volatile composition is observed.

The techniques for the extraction of volatile oils have also been reported to cause variations in the volatile compounds of samples [20]. The techniques used to extract volatile compounds from mango include solvent extraction [15], vacuum steam distillation followed by solvent extraction [21], simultaneous distillation-extraction [1,5,6], static headspace [22,23], solid-phase extraction [16], headspace solid-phase microextraction [19], and so on. The studies of Larrauri *et al.* [24] showed that mango peel fibers can be included in high dietary fiber diets because the fibers have beneficial health effects. 

HD is the most common approach to the extraction of essential oils from medicinal herbs and plants, as described in various pharmacopoeias, such as the 2005 *Chinese Pharmacopoeia* [25]. Conventional methods present several drawbacks such as long extraction times, potential loss of volatile constituents, high energy use, and so on. Thus, developing an alternative extraction technique that is rapid, sensitive, safe, and energy-efficient is highly desirable. New extraction techniques have been applied to shorten extraction time, reduce organic solvent consumption, improve extraction yield, enhance extract quality, prevent pollution, and reduce sample preparation costs; these techniques include microwave assisted extraction (MAE), supercritical fluid extraction (SFE), and ultrasonic assisted extraction (UAE) [26,27]. Some recently published studies have successfully used a microwave oven as a heater in the extraction of essential oils from medicinal plants and herbs [1,28,29,30]. To take advantage of microwave heating with traditional HD, MAHD was subsequently developed and used for the extraction of essential oils from *Dictyopteris membranacea*, *Salvia rosifolia* Sm., *Thymus vulgaris* L, *Cuminum cyminum* L., and *Zanthoxylum bungeanum* Maxim [20,27,28,29].

To the best of the authors’ knowledge, however, no data on the MAHD of volatiles in mango flowers have been reported. Therefore, the objective of the present work is to investigate the potential of MAHD for extracting essential oils from mango flowers. The compositions of the essential oils extracted by MAHD and HD were analyzed by GC-FID and GC-MS. The extraction time, volatile components, and extraction yield of MAHD were also compared with those of HD.

## 2. Results and Discussion 

Essential oils from mango flowers were obtained by MAHD and HD. The oils were subsequently analyzed by GC-FID and GC-MS. The identities of the extracted essential oils from both methods are listed in Table 1. 

The yields of the oils (w/w %) at extraction were in the following order: MAHD (0.16%) > HD (0.11%). In total, 51 and 56 constituents were identified and quantified in MAHD and HD extraction, respectively (Table 1).

The results of GC-MS analyses show that a major portion of the mango flowers is composed of monoterpene hydrocarbons (Table 1). As for the oil extracted by MAHD, the main constituents were terpinolene (43.17%), δ-3-carene (7.72%), limonene (5.22%), α-terpinene (4.64%), and *p*-cymen-8-ol (4.04%). The main content in the essential oil was monoterpene hydrocarbons (74.12%), followed by sesquiterpene hydrocarbons (9.86%), oxygenated monoterpenes (9.49%), “others” (2.63%), and oxygenated sesquiterpenes (0.30%), as shown in Table 1.

In the oil extracted by HD, the main constituents were terpinolene (50.16%), δ-3-carene (8.17%), limonene (5.39%), α-terpinene (5.31%), and β-selinene (3.75%). The essential oil was composed mainly of monoterpene hydrocarbons (80.56%). The percentage of sesquiterpene hydrocarbons, oxygenated monoterpenes, oxygenated sesquiterpenes, and hydrocarbons, such as alkanes and esters (classified as “others”), were 12.5%, 4.80%, 1.08%, and 0.4%, respectively (Table 1). 

The constituents of the essential oils extracted by both MAHD and HD were similar. Both essential oils were found to be rich in monoterpene hydrocarbons. terpinolene (compound 20) was the most abundant component in mango essential oil (43.17% for MAHD and 50.16% for HD) followed by p-δ-3-carene (compound 11), limonene (compound 15), α-terpinene (compound 12) and β-Selinene (compound 51). These results are in good agreement with [8], who reported 20 mango cultivars which were investigated by means of simultaneous distillation-extraction, GC, and GC-MS. The main compounds of essential oil of mango from Kenya were α-pinene, α-phellandrene, limonene and *p*-cymene [9], while α-pinene, sabinene, β-pinene and limonene were dominant components in the fruit oil of mango from India [10]. The dominant compounds mango leaf oil from Nigeria were δ-3-carene, α-gurjunene, β-selinene and β-caryophyllene, while fruit peel oil yielded mainly δ-3-carene and α-pinene; whereas according reference 12, the leaf oil of mango form Nigeria possessed a considerable proportion of α-gurjunene, β-selinene, β-caryophyllene and α-humulene. 

The appearance, specific gravity, and refractive index of the essential oils extracted with HD and MAHD are provided in Table 2. The specific gravities and refractive indices of the essential oils are similar, with the only difference being that the color of the MAHD essential oils was lighter than that of the HD oils. Thus, MAHD does not present any problems in the extraction of essential oils from mango flowers.

## 3. Experimental 

### 3.1. Plant material

Fresh mango flowers were collected from Southern regions of China on 19 December 2008. The plant was identified by Vice Professor Xiongwei Chen of the College of Life Sciences at Zhaoqing University. Voucher specimens (code number 0826) were deposited at the herbarium of the College of Life Sciences, Zhaoqing University.

### 3.2. Isolation of essential oils

Hydrodistillation was performed according to the method recommended in the *Chinese Pharmacopoeia* [25]. Fresh mango flowers (500 g) were hydrodistilled for 4 h using a Clevenger-type apparatus to yield 0.11% of a yellowish, strong-smelling oil. 

Fresh flowers (200 g) were hydrodistilled at 800 W for 75 min using an adapted microwave distillation apparatus (wp800, Galanz, China), which consists of a microwave oven connected to a Clevenger-type apparatus to yield 0.16% of a yellowish, strong-smelling oil.

The extracted essential oils were dried over anhydrous sodium sulfate and stored in amber vials at 4 °C until analysis. The oil was subsequently subjected to GC-FID and GC-MS analyses, and then dissolved in *n*-hexane (10% v/v) before its composition was chromatographically determined.

### 3.3. GC and GC-MS analyses

The mango essential oils were analyzed by GC using a GC-2010 with AOC-20i auto injection system (Shimadzu Ltd., Japan) system and a silica capillary Rtx-5ms column (30 m × 0.25 mm i.d.; film thickness, 0.25 μm) with nitrogen at 1 mL/min. Initial oven temperature was 80 °C for 3 min, increased to 6 to 150 °C/min. The temperature was then kept constant at 150 °C for 16 min and increased to 30 to 200 °C/min. Subsequently, the temperature was kept constant at 200 °C for 2 min and increased to 20 to 240 °C for 2 min. Injector and detector temperatures were 220 and 280 °C, respectively. The carrier gas was nitrogen (volume, 1.0 µL), injected at a flow rate of 1.0 mL/min at constant flow. A sample was injected auto without dilution. The percentages of the constituents were calculated by the electronic integration of the FID peak areas without the use of response factor correction. 

GC-MS analyses were performed with a GC-MS QP2010 Plus with AOC-20i auto injection system (Shimadzu Ltd., Japan) system and a silica capillary Rtx-5ms column (30 m × 0.25 mm i.d.; film thickness, 0.25 μm) with helium. GC oven temperature conditions were as described above. Split flow was adjusted to 50 mL/min, with an injector temperature of 280 °C. The carrier gas was helium, passed at a flow rate of 1.0 mL/min. A diluted sample (1.0 µL, 1/10 in ether) was autoinjected. The mass spectra were recorded at 70 eV, and the mass range was *m*/*z* 35 to 425. The ion source and interface temperatures were 200 and 280 °C, respectively. 

### 3.4. Identification of components

The constituents of the essential oils were identified by calculating their retention indices under temperature-programmed conditions for *n*-alkanes (C_6_–C_24_) and oils on an Rtx-5ms column under the same chromatographic conditions as those in Section 2.3 Individual compounds were identified by comparing their mass spectra with those of the internal reference mass spectra library or with authentic compounds. The compounds were then confirmed by comparing their retention indices with the NIST 2005 GC-MS library data on the GC-MS Adams library spectra [1]. For quantification purposes, the relative area percentages obtained by FID were used without correction factors.

### 3.5. Physical properties

The physical properties, such as appearance, refractive index, specific gravity, and color, of the essential oils extracted (by both methods) from the flowers of the mango samples were determined using the Food Chemical Codex method [31]. The refractive index and specific gravity were measured at 20 °C. 

## 4. Conclusions 

Both MAHD and conventional HD are based on the same process as essential oil-water heteroazeotrope. Therefore, oils extracted using both methods must contain a large amount of high volatility compounds, and their chemical composition should, theoretically, be similar. Microwave energy may improve the release of the volatile compounds from the matrix, and high energy flux may enhance their co-distillation. As previously shown by scanning electron microscopy and the cytochemistry of lavender glandular trichomes [32], microwaves cause the glandular walls to quickly rupture, resulting in high extraction efficiency at a shorter time. The results of the GC-MS analyses indicate that no significant differences between the essential oils extracted by MAHD and those extracted by HD were found. MAHD offered substantial advantages over conventional HD. As an excellent alternative to HD, MAHD may have no adverse effects on the composition of extracted essential oils. MAHD is also more environment-friendly than HD. Compared with many solvent extraction techniques, such as Soxhlet, solvent extraction, and accelerated solvent extraction, MAHD is a modern, green, and rapid approach.

## Figures and Tables

**Table 1:** Constituents of essential oils from the flowers of mango extracted with MAHD and HD.

Peak number	Compound	Formula	RI	% Composition		Methods of Identification
				MAHD	HD	
1	trans-3-Hexen-1-ol	C6H12O	858	0.09	0.14	RI, MSb
2	1-Hexanol	C6H14O	878	0.06	0.10	RI, MS
3	Heptanal	C7H14O	903	0.05	0.06	RI, MS
4	α-Pinene	C10H16	932	3.47	2.39	RI, MS, CoI
5	Camphene	C10H16	945	0.40	0.27	RI, MS
6	Sabinene	C10H16	975	0.14	0.07	RI, MS
7	Myrcene	C10H16	978	2.23	2.11	RI, MS
8	β-Pinene	C10H16	979	1.27	0.95	RI, MS
9	2-Carene	C10H16	995	1.04	1.01	RI, MS
10	α-Phellandrene	C10H16	1001	1.68	1.76	RI, MS
11	d-3-Carene	C10H16	1011	7.72	8.17	RI, MS
12	α-Terpinene	C10H16	1017	4.64	5.31	RI, MS
13	o-Cymene	C10H14	1022	0.88	-a	RI, MS
14	p-Cymene	C10H14	1026	-	0.34	RI, MS
15	Limonene	C10H16	1029	5.22	5.39	RI, MS
16	(Z)-β-Ocimene	C10H16	1040	1.10	1.25	RI, MS
17	(E)-β-Ocimene	C10H16	1050	0.25	0.23	RI, MS
18	γ-Terpinene	C10H16	1059	0.69	0.91	RI, MS
19	(E)-Linalool oxide	C10H18O2	1070	0.06	0.25	RI, MS, CoI
20	Terpinolene	C10H16	1084	43.17	50.16	RI, MS
21	Thujol	C10H18O	1095	0.43	0.06	RI, MS
22	Linalool	C10H18O	1100	0.48	0.63	RI, MS
23	Hotrienol	C10H16O	1101	0.71	1.09	RI, MS
24	4-Isopropyl-1-methyl-2-cylohexen-1-ol	C10H18O	1117	-	0.13	RI, MS
25	(3E,5E)-2,6-Dimethyl-1,3,5,7-octatetraene	C10H14	1134	0.22	0.24	RI, MS, CoI
26	cis-Verbenol	C10H16O	1140	0.82	-	RI, MS
27	(Z)-2-Nonenal	C9H16O	1145	0.06	0.05	RI, MS
28	2-Ethylcyclohexanone	C8H14O	1158	0.46	-	RI, MS
29	p-Mentha-1,5-dien-8-ol	C10H16O	1159	0.06	-	RI, MS, CoI
30	Terpinen-4-ol	C10H18O	1178	0.85	0.82	RI, MS
31	Nerol oxide	C10H16O	1162	0.07	0.11	RI, MS
32	p-Cymen-8-ol	C10H14O	1182	4.04	0.33	RI, MS
33	α-Terpineol	C10H18O	1190	0.75	0.97	RI, MS
34	2-Methylisoborneol	C11H20O	1197	1.13	-	RI, MS
35	γ-Terpineol	C10H18O	1199	0.15	0.18	RI, MS
36	Nerol	C10H18O	1233	0.09	0.11	RI, MS
37	Geraniol	C10H18O	1255	0.05	0.06	RI, MS
38	trans-3-Caren-2-ol	C10H16O	1272	0.18	-	RI, MS
39	p-Ethylguaiacol	C9H12O2	1287	0.74	-	RI, MS
40	α-Copaene	C15H24	1375	0.24	0.25	RI, MS
41	β-Elemene	C15H24	1391	0.05	0.11	RI, MS
42	Methyl eugenol	C11H14O2	1401	0.04	0.05	RI, MS, CoI
43	α-Gurjunene	C15H24	1412	1.17	1.50	RI, MS
44	β-Caryophyllene	C15H24	1420	1.34	1.84	RI, MS
45	Carvone hydrate	C10H16O2	1424	0.75	0.06	RI, MS
46	Aromadendrene	C15H24	1443	0.15	0.17	RI, MS
47	α-Guaiene	C15H24	1453	0.28	1.29	RI, MS
48	α-Humulene	C15H24	1453	0.71	0.82	RI, MS
49	Alloaromadendrene	C15H24	1461	0.16	-	RI, MS
50	γ-Muurolene	C15H24	1475	0.28	-	RI, MS
51	β-Selinene	C15H24	1482	3.01	3.75	RI, MS
52	α-Amorphene	C15H24	1484	-	0.37	RI, MS
53	Germacrene D	C15H24	1487	0.77	0.86	RI, MS
54	α- Muurolene	C15H24	1499	0.27	0.37	RI, MS
55	δ-Cadinene	C15H24	1519	0.30	0.46	RI, MS
56	Epiglobulol	C15H26O	1532	0.18	0.68	RI, MS
57	δ-Guaiene	C15H24	1526	0.51	0.59	RI, MS, CoI
58	Ledol	C15H26O	1552	-	0.11	RI, MS
59	Guaia-3,9-diene	C15H24	1556	0.62	0.12	RI, MS
60	Caryophyllene oxide	C15H24O	1573	0.12	-	RI, MS
61	α-Cadinol	C15H26O	1653	-	0.29	RI, MS
Total				96.40	99.34	
Monoterpene hydrocarbons				74.12	80.56	
Oxygenated monoterpenes				9.49	4.8	
Sesquiterpene hydrocarbons				9.86	12.5	
Oxygenated sesquiterpenes				0.30	1.08	
Othersc				2.63	0.40	
Oil yield (w/w%)				0.16	0.11	
Types				56	51	

^a^ absent. RI, retention indices relative to C_6_–C_24_ *n*-alkanes on the Rtx-5ms column; ^b^ MS, mass spectrum; CoI, coinjection with an authentic sample. ^c^ Others: hydrocarbons such as alkanes and esters.

**Table 2:** Physical properties of essential oils. ^d^

Physical properties	MAHD	HD
Refractive index	1.487	1.487
Specific gravity	0.921	0.917
Appearance	Pale yellow	Yellow

^d^ The refractive index and specific gravity were measured at 20 °C.
